# Reviewed article: Silva LAN, Delpino FM, Figueiredo LM, Thumé E, Tomasi E, Facchini LA, Nunes BP. Adult use of health services: a cross-sectional population-based study, Pelotas, Brazil, 2012 and 2021. Epidemiol Serv Saude. 2026:35;e20250460

**DOI:** 10.1590/S2237-96222026v35e20250460.c

**Published:** 2026-04-20

**Authors:** Sandra Maria do Valle Leone de Oliveira

**Affiliations:** 1 Fundação Oswaldo Cruz, Campo Grande, MS, Brazil Fundação Oswaldo Cruz Campo Grande MS Brazil

## First round comments

O artigo possui potencial para publicação, mas requer revisão substancial na metodologia e nas análises. A ausência de modelos ajustados e explicações adequadas sobre o delineamento amostral compromete a robustez dos achados e sua interpretação. Os autores devem responder às questões metodológicas levantadas pelos pareceristas, ajustar inconsistências analíticas e incluir informações faltantes. A clareza na apresentação dos resultados e a coerência com a discussão também devem ser aprimoradas.

Reescrever e detalhar o processo amostral, incluindo: Se foi amostragem probabilística em 2012;Se todos os setores censitários foram elegíveis;Como foram tratados os domicílios recusados.Explicitar o período de referência para a pergunta sobre uso do serviço de saúde.

2-Justificar e revisar a categorização racial; preferencialmente apresentar dados desagregados.

3-Incluir análise multivariada (regressão logística ou Poisson com variância robusta), para ajustar os efeitos das variáveis entre si ou justificar a não utilização.

4-Justificar as análises não ponderadas de 2021 e discutir suas implicações.

5-Revisar e explicar o uso da expressão “duplas estratificações”.

6-Inserir dados sobre cobertura de planos de saúde no município.

Adicionar limitações específicas, como:

7-Comparabilidade limitada entre amostras;

8-Potencial viés de seleção;Análises sem controle de confundimento.

Recommendation: Major revisio

## Second round comments

O manuscrito é sólido, bem ancorado metodologicamente e pertinente para a Saúde Coletiva. Sugiro uma inclusão explícita dos valores do SII (± RII), clarificações de comparabilidade e pequenos acertos editoriais. 

Sugiro:

1-Inserir, no Resumo/Resultados, os valores do SII com IC95% em 2012 e 2021 e explicitar se houve teste de interação ano×classe (e/ou ano×escolaridade), com p-valor. Seria importante acrescentar números no texto e legenda da Figura/Tabela correspondente.

2- Deixar explícito no Métodos que 2012 analisou ≥20 anos (embora elegíveis ≥10) e 2021 ≥18; justificar a opção ou apresentar sensibilidade harmonizando a idade (≥20).

3- Quanto a ponderação/ análise sem peso (2021) você já indica que foram exploratórias. Reforce numericamente o impacto (Δ p.p. e variação das RPs) e mantenha claro que todas as conclusões derivam das análises ponderadas com 1-2 frases adicionais nos Resultados.

3-Em um trecho há menção a “leve redução”; em outro, “aumentou, porém não significativo”. Uniformizar a mensagem segundo os valores do SII. Por exemplo, manter a forma “aumentou sem significância em ambos os anos” (ou a alternativa correta, com números).

4-Denominadores em Tabela 3, por favor, tente deixar explícito se as proporções reportadas são composição dos usuários de cada natureza de serviço ou prevalência dentro do estrato. Por exemplo, use uma nota de rodapé esclarecendo o denominador.

Reveja detalhes editoriais:

1- Terminologia: “inquéritos transversais repetidos”; “EAI Pelotas”.

2-Linguagem: “foi avaliada por meio”, “conforme descrito”.

3-Padronização numérica (RESS): vírgula para decimais; IC95% x,x; y,y; p-valor com 3 casas (usar “<0,001” quando aplicável).

4-Corrigir typos: “IC95%” (substituir “IC9%”), incluir “%” ausentes.

5-Raça/cor: grafar “raça/cor da pele”; ABEP com nota de rodapé.

Recommendation: Minor revision

